# Influence of transducer types on bone conduction hearing thresholds

**DOI:** 10.1371/journal.pone.0195233

**Published:** 2018-04-11

**Authors:** Laura Fröhlich, Stefan K. Plontke, Torsten Rahne

**Affiliations:** University Hospital Halle (Saale), Department of Otorhinolaryngology, Head and Neck Surgery, Martin Luther University Halle-Wittenberg, Halle, Germany; University of Göttingen, GERMANY

## Abstract

**Objective:**

Different types of bone conduction transducers with different physical and electro-acoustic properties are available for audiometric hearing threshold measurements. The reference equivalent threshold vibratory force levels (RETVFL) specified in ISO 389–3 are based on measurements conducted with the B71 and KH70 transducers but apply to all types of transducers available for bone conduction audiometry. The objective of this study was to compare bone conduction hearing thresholds measured by different transducers.

**Design:**

In a prospective study the hearing thresholds were measured psychometrically between 125 Hz and 8000 Hz using the Radioear B71, B81 and Präcitronic KH70 transducers.

**Study sample:**

Twenty-one normal hearing participants and fifteen hearing impaired participants.

**Results:**

In both groups significant differences were found between the thresholds measured with the different transducers at the low frequencies 125 Hz and 250 Hz and the high frequencies 3000 Hz, 4000 Hz, 6000 Hz and 8000 Hz. In the normal hearing group, deviations from the reference threshold 0 dB HL towards lower thresholds were observed for the B71 and B81 at 125 Hz and at the high frequencies 3000 Hz, 4000 Hz, 6000 Hz and 8000 Hz.

**Conclusions:**

RETVFL-values should be reassessed and provided specifically for the different transducers.

## Introduction

In bone conduction (BC) audiometry different types of BC transducers, which have different physical and electro-acoustic properties, can be used for hearing threshold measurements. The recently introduced BC transducer type B81 (Radioear, New Eagle, USA) was found to generate higher output levels and less distortion than the widespread type B71 (Radioear) for frequencies up to 1500 Hz [1]. Also the KH70 (Präcitronic, Dresden, Germany), which is no longer manufactured as of 2006, is still used in many facilities due to its low distortion [2]. Physically, the B71 and B81 are very similar. Both transducers are placed in a plastic housing, the length is approximately 32 mm, and the width is approximately 18 mm. The height of the B71 is about 19 mm, the B81 is 16 mm high. The weight of both devices is 20 g. However, the height of the KH70 is approximately 2.5 times that of the B71 and B81 and its weight is 96 g. Moreover, the KH70 is encapsulated in a rubber housing. Despite these differences between the transducers, the BC hearing thresholds determined by means of different transducers at different facilities have to be comparable. Otherwise hearing threshold results and diagnoses in the same person would differ depending on where they were confirmed, i.e., depending on which type of transducer was used. This would have an impact on the method of treatment including the choice of hearing aids.

Therefore, reference zeros for the calibration of audiological equipment, such as the reference equivalent threshold vibratory force levels (RETVFL) for BC transducers, are specified in standards and International Electrotechnical Commission (IEC) specifications [3, 4]. The RETVFLs, i.e., the BC hearing 0 dB HL references for each frequency, are based on the means of hearing thresholds measured in three independent investigations on normal hearing individuals with the B71 and KH70 BC transducers. In two of these studies the B71 was used at the frequencies 250 Hz, 500 Hz, 1000 Hz, 2000 Hz, 3000 Hz and 4000 Hz [5, 6]. In the other study the KH70 was used and the test frequencies were 125 Hz, 250 Hz, 500 Hz, 750 Hz, 1000 Hz, 1500 Hz, 2000 Hz, 3000 Hz, 4000 Hz, 5000 Hz, 6000 Hz, 6300 Hz and 8000 Hz [7]. This single series of equivalent threshold force levels, i.e., the means of the thresholds obtained in the three studies with the B71 and the KH70, is valid for the BC hearing threshold for any transducer which is commercially available [8].

How BC hearing thresholds are influenced by the type of transducer has been investigated on normal hearing participants in previous studies. Brinkmann and Richter [2] measured BC hearing thresholds with the B71 and KH70 between 125 Hz and 8000 Hz. Good agreement between the thresholds obtained with the different transducers was found and it was concluded that the reference hearing thresholds can be described by two series of equivalent threshold force levels, one for the mastoid and one for the forehead position for all BC transducers. However, Sauer and Bellack [9], who also measured BC hearing thresholds with the KH70, reported lower thresholds with the KH70 at 125 Hz and 8000 Hz compared to the results of Brinkmann and Richter [9]. Another study was conducted by Frank et al. [10] who included the B71 and KH70 transducers at frequencies between 250 Hz and 4000 Hz. Frank et al. observed that the thresholds measured with the KH70 at 250 Hz were approximately 10 dB higher than with the B71 and about 5 dB lower at 500 Hz. The authors concluded that the RETVFL should be specified by BC transducer type. BC threshold measurements with different transducers on hearing impaired participants are not reported in the literature.

These results from the literature show that the reliability of BC hearing thresholds obtained with different transducers is a contentious issue. The purpose of this study was to systematically measure hearing thresholds with the conventional B71, the new B81, and the KH70 types of BC transducers and compare the results. The measurements were performed on a group of normal hearing (NH) and a group of hearing impaired (HI) participants.

## Methods

### Participants

The participants in this study signed an informed consent form before the onset of the experimental procedures. The study protocol, laboratory protocols, informed consent form, and participant information sheets were submitted to the Ethical Review Board of the Martin Luther University Halle-Wittenberg and written approval was given for the study (approval number: 2015–143).

In the NH group, 21 adults (13 male and 8 female) participated aged between 20 and 32 years (mean 25.5 years). The inclusion criteria were for air conduction (AC) hearing thresholds to not exceed 10 dB HL at the frequencies 125 Hz, 250 Hz, 500 Hz, 750 Hz, 1000 Hz, 1500 Hz, 2000 Hz, 3000 Hz, 4000 Hz and 15 dB HL at the higher frequencies 6000 Hz and 8000 Hz.

The HI group consisted of 15 participants (9 male and 6 female) aged between 47 and 81 years (mean 66.6 years). All participants were patients of the University Hospital Halle (Saale), so that their pure tone audiograms already existed. The inclusion criteria were for BC thresholds to be 15 dB HL or worse in all audiometric BC test frequencies in the existing clinical audiogram 250 Hz, 500 Hz, 750 Hz, 1000 Hz, 1500 Hz, 2000 Hz, 3000 Hz, 4000 Hz, and 6000 Hz.

### BC transducers

Before commencement of the study, the frequency response and total harmonic distortion of the B71, B81, and KH70 were measured in order to verify the functionality of the transducers. The set-up and procedure used to obtain these data are described by Fredén Jansson et al. [1].

### Procedure

All measurements were conducted in an anechoic chamber, where the ambient sound pressure levels did not exceed the permissible levels specified in ISO 8253–1 [4]. In the NH group, the participants’ AC pure tone audiograms were measured at 125 Hz, 250 Hz, 500 Hz, 750 Hz, 1000 Hz, 1500 Hz, 2000 Hz, 3000 Hz, 4000 Hz, 6000 Hz, and 8000 Hz with an AT900 clinical audiometer (Auritec GmbH, Germany) and HDA 200 headphones (Sennheiser electronic GmbH and Co. KG, Germany) to check if the inclusion criteria were met. For the HI participants, clinical audiograms already existed and they were chosen to participate in the study based on the BC thresholds in these audiograms.

The experimental set-up consisted of the transducers connected to the sound field output of an SD-50 audiometer (Siemens GmbH, Germany) in order to increase the output levels compared to the conventional BC output. No additional amplifier was used. The BC hearing thresholds were then measured psychometrically between frequencies of 125 Hz and 8000 Hz for mastoid coupling using pulsed warble tones. The transducers were coupled to the mastoid by headbands made of spring steel. The KH70 comes along with a headband which is provided with a setting screw that allows the adjustment to the individual head size, whereas the B71 and B81 were used with the Radioear P333 headband (Radioear, New Eagle, USA). The headbands are designed to couple the BC transducers to the head with a static force of 5.4 N ± 0.5 N. The presentation level was increased in steps of 5 dB until the participant responded to hear the tone. Afterwards, the level was decreased by 15 dB so that the test tone was inaudible. Then the presentation level was increased again in steps of 1 dB. The procedure was repeated until the participant repeatedly answered to hear the tone at the same level for three times. This level was defined as the BC hearing threshold. The procedure was conducted using the B71, the B81, and the KH70 in a randomized order. The BC transducer left a mark on the participant’s skin so that the following transducer could be placed at the exact same position as the previous one. This ensured an identical stimulation site for each transducer. The better ear was chosen as the test side for the NH participants, so that no masking was applied. For the HI participants, based on the clinical audiograms the worse side was chosen as the test side determined by the BC 4-PTA (the four-frequency pure tone average at 0.5, 1, 2, 4 kHz). The clinical audiograms were obtained with the KH70 transducer as this is the standard used for BC threshold testing at the University Hospital Halle. If there were unmeasurable thresholds due to exceedance of the audiometer limit, the better side was chosen as the test side. The purpose of the procedure was to ensure obtaining BC thresholds at preferably high presentation levels. The purpose of the study was not to measure the participants’ actual hearing threshold but to compare the results among transducers. Therefore, no masking was applied in both experimental groups and the thresholds were measured independently from the actual perception side.

### Statistical analysis

The mean thresholds were statistically analyzed by parametric statistics as normal distribution of the data was confirmed by a Kolmogorov-Smirnov test. For the NH group, dependent *t*-tests were used to compare the BC thresholds with the AC thresholds. The level of significance was reduced to 0.002 for multiple comparisons using Bonferroni correction (11 frequencies×3 transducers = 33 *t*-tests, *p* = 0.05/33 = 0.002). A two-way analysis of variance (ANOVA) for repeated measures analyzed the results with respect to the study objective, i.e., the effect of the within-subject factors *transducer* (B71, B81 and KH70) and *frequency* (all 11 test frequencies between 125 Hz and 8000 Hz) on the BC thresholds. For significant effects post hoc *t*-tests were used to further analyze the results. The level of significance was reduced to 0.002 in the *t*-tests for multiple comparisons as explained above.

The results from the HI group were analyzed by paired *t*-tests due to missing data from some participants at some frequencies (see results section).

### Calibration

Since the experimental set-up consisted of the transducers connected to the sound field output of an SD 50 audiometer, it was necessary to calibrate the threshold levels obtained in the experiments. The transducers were attached to an artificial mastoid B&K 4930 (Brüel & Kjær Sound & Vibration Measurement A/S, Denmark) with a static force of 5.4 N. The output voltage level from the artificial mastoid *V*_out_ was measured by a sound level meter B&K 2235 (Brüel & Kjær Sound & Vibration Measurement A/S, Denmark) in dB for each test frequency. The voltage level was converted into the corresponding force level in dB relative to 1μN by considering the pad correction function *P*(*jω*) = *V*_out_/*F*_in_ of the artificial mastoid. The force level was then converted into hearing levels (dB HL) using the RETVFLs from ISO 389–3. Thus, the actual corresponding hearing level for the respective audiometer level was known for each transducer at each test frequency which represents the calibration of the transducers connected to the sound field output. Due to the linearity of the audiometer it was possible to convert any audiometer level into the actual hearing level. This conversion was done after the threshold recordings.

## Results

**Fig 1:**
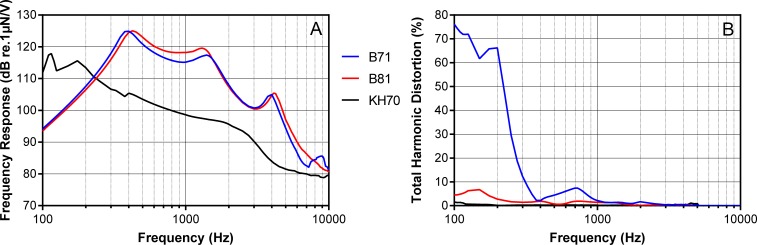
Frequency response and total harmonic distortion of the B71, B81 and KH70 transducers. The frequency response of the B71, B81 and KH70 in dB relative to 1μN/V obtained at an input voltage of 1V_RMS_ (A) and the total harmonic distortion of the transducers for an input voltage of 1V_RMS_.

In Fig 1 the electro-acoustic characteristics of the devices are depicted. Panel A shows the frequency response of the B71, B81 and KH70 in dB relative to 1μN/V obtained at an input voltage of 1V_RMS_ and Panel B shows the total harmonic distortion of the transducers while they were driven by an input voltage of 1V_RMS_.

All data underlying the findings of this study, i.e., the BC threshold values measured in this study on normal hearing and hearing impaired participants, can be found in S1 Table.

### Hearing thresholds of normal hearing participants

**Fig 2:**
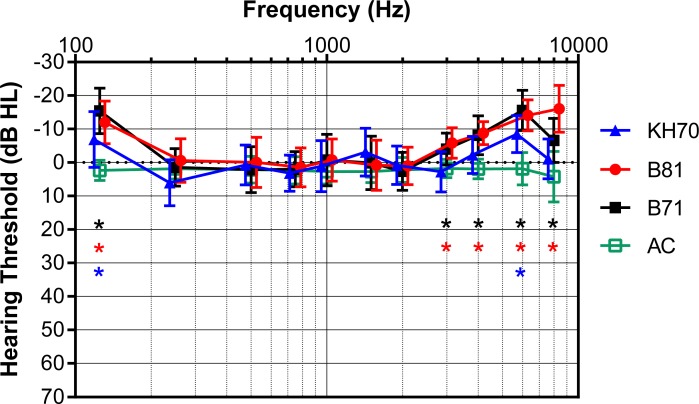
Mean BC hearing thresholds of SH participants measured with the B71, B81 and KH70 transducers. The mean BC hearing thresholds measured on NH participants with the B71, B81 and KH70 are shown. Also the AC thresholds are shown and significant differences between the BC and AC thresholds are indicated for each transducer type(*, all *p*s<0.002).

Fig 2 shows the mean AC and BC hearing thresholds with the different transducers on the NH participants. While the mean AC thresholds form a straight line close to 0 dB HL as it is expected for NH participants, the BC thresholds obtained with the B71 and B81 were around 0 dB HL between 250 Hz and 2000 Hz but lower (better) at 125 Hz and frequencies above 2000 Hz. This results in a curved shape of the BC audiogram. Dependent *t*-tests showed that at the frequencies 125 Hz, 3000 Hz, 4000 Hz, 6000 Hz and 8000 Hz, the BC thresholds measured with the B71 and B81 were significantly lower than the AC thresholds. With the KH70 the BC thresholds were significantly lower than the AC thresholds at 125 Hz and 6000 Hz (*, all *p*s<0.002).

Regarding the influence of the BC transducer, the two-way repeated measures ANOVA showed a significant effect of the *transducer* on the mean hearing thresholds (*F*(1.4, 28.7) = 34.3, *p*<0.05) as well as a significant effect of the *frequency* (*F*(4.9, 98.5) = 29.6, *p*<0.05). Also the interaction *transducer* × *frequency* was significant (*F*(8.3, 166.3) = 20.5, *p*<0.05). Since the assumption of sphericity was violated for the factors *transducer* (χ^2^(2) = 9.43, *p*<0.05) and *frequency* (χ^2^(54) = 93.01, *p*<0.05) as well as the interaction *transducer* × *frequency* (χ^2^(209) = 420.37, *p*<0.05), the degrees of freedom were corrected using the Greenhouse-Geisser correction (ε<0.75, respectively). Post hoc *t*-tests showed significant differences (all *p*s<0.002) between the B71 and B81 at 125 Hz, where the threshold obtained with the B71 was significantly lower than with the B81, and at 8000 Hz, where the threshold was significantly higher with the B71. However, comparing the B71 and B81 with the KH70, the hearing thresholds obtained by the KH70 were significantly higher than with the B71 and the B81 at 125 Hz, 250 Hz, and at frequencies above 3000 Hz. However, at 1500 Hz, the threshold measured with the KH70 transducer was significantly lower than with the B81. This is illustrated in Table 1 which shows the differences between the hearing thresholds measured with the B71, B81 and KH70 on the NH group.

**Table 1:** Differences between BC hearing thresholds measured with the B71, B81 and KH70 transducers.

Frequency (Hz)	Differences between BC hearing thresholds (dB HL)					
	B71-B81		B71-KH70		B81-KH70	
	NH	HI	NH	HI	NH	HI
125	**-3**	**-5**	**-8**	**-10**	**-5**	-5
250	2	1	**-5**	**-8**	**-7**	**-10**
500	2	-1	1	3	-1	3
750	1	0	-1	1	-2	1
1000	0	-1	-2	-2	-2	-1
1500	-1	-1	3	**10**	**4**	**11**
2000	2	1	2	3	0	2
3000	2	2	**-7**	-5	**-9**	**-7**
4000	1	0	**-6**	**-5**	**-7**	**-5**
6000	-2	-2	**-7**	-6	**-5**	-4
8000	**9**	**11**	**-6**	-2	**-15**	**-12**

Average differences between the BC hearing thresholds measured with the B71, B81 and KH70 for NH and HI participants.

**Bold**: significant differences (all ps<0.002)

### Hearing thresholds of hearing impaired thresholds of hearing impaired participants

**Fig 3:**
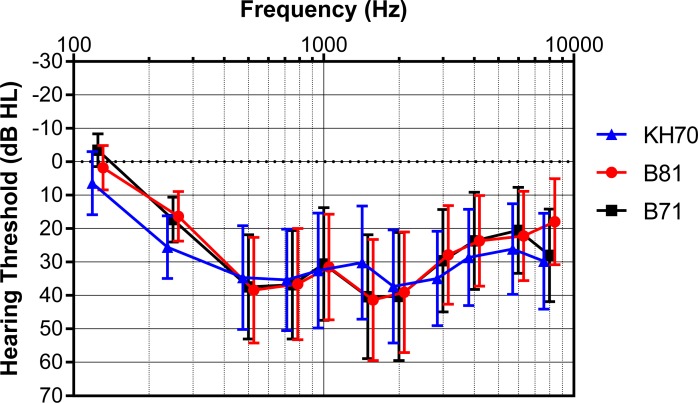
Mean BC hearing thresholds of SH participants measured with the B71, B81 and KH70 transducers. The mean BC hearing thresholds measured on SH participants with the B71, B81 and KH70 are shown in an audiogram.

The results from the measurements on HI participants are similar to those from the NH participants. However, the BC hearing thresholds exceeded the measurement limit of the transducers at some frequencies for some participants. In order to be able to compare the results of the different transducers only those thresholds were considered for a participant at certain frequencies if the thresholds were measurable with all transducers. For instance, if a threshold was measurable at 3000 Hz with the B71 and B81 but unmeasurable with the KH70 due to the lower maximum output levels of the KH70, the data obtained with the B71 and B81 at 3000 Hz was omitted. The number of HI participants who had measurable thresholds with all transducers was 12 at 125 Hz and 250 Hz, 15 between 500 Hz and 2000 Hz, 13 at 3000 Hz, 12 at 4000 Hz, 11 at 6000 Hz, and 10 at 8000 Hz. In 8 of the 15 measurements the better side has to be chosen (see Methods section) instead of the worse side. The results are illustrated in Fig 3, showing differences between the transducers similar to those observed for the NH participants. The *t*-tests showed significant differences between the B71 and B81 only at 125 Hz where the threshold was lower with the B71 and at 8000 Hz where the threshold was lower with the B81. Comparing the B71 to the KH70, significantly lower thresholds were found for the B71 at 125 Hz, 250 Hz, and 4000 Hz but significantly higher thresholds at 1500 Hz. Similarly, for the B81 in comparison to the KH70, significantly lower thresholds were found with the B81 at 250 Hz, 3000 Hz, 4000 Hz and 8000 Hz, whereas at 1500 Hz the thresholds were significantly higher than with the KH70.

## Discussion

The results show statistically significant differences ranging from 4 to 15 dB (see Table 1) between the mean BC thresholds obtained with the different transducers. Although the differences are small for some frequencies and transducer comparisons, there are also differences exceeding the typical 5 dB audiometric step size so that they should be considered as clinically relevant. This is because the audiometric results have an impact on the method of a patient’s treatment including the choice of hearing aids. For example, the indication criteria for BC hearing aids or implants can be very strict and BC threshold testing should be accurate. With the B71 and B81, which are similar in design, shape and weight, differences were found in both the NH and the HI group at 125 Hz and 8000 Hz. Comparing the B71 and B81 with the KH70, the results from both experimental groups indicate a deviation towards significantly lower (better) thresholds with the B71 and B81 at 125 Hz and 250 Hz and frequencies above 3000 Hz. This is in line with Frank et al. [10] who found significantly lower thresholds with the B71 than with the KH70 at low frequencies. No frequencies above 4000 Hz were tested in the study conducted by Frank et al., so that deviations for frequencies 6000 Hz and 8000 Hz observed in this study are the first to be reported.

An influence of the coupling force on the results is unlikely. The coupling force was controlled by using standard headbands (P333 and the KH70 headband) which are designed to couple the transducers to the head with a static force of 5.4 N ± 0.5 N. Deviations of the BC hearing thresholds resulting from BC transducer coupling forces outside the range of 5.4 N ± 0.5 N can only occur for very small or large heads. These deviations would be systematic though, as the results from the transducers were compared, i.e., the difference between the BC thresholds obtained with different transducers was the objective of the study. Each of the three transducers was tested in one participant, respectively (repeated measure).

Instead, the different electro-acoustic properties of the transducers might be responsible for the deviations between the results obtained with the different transducers and the deviations from the AC hearing thresholds of NH participants towards lower thresholds. At low frequencies, harmonic distortion is a problem, while at high frequencies acoustic radiation could have influenced the results. Eichenauer et al. [11] measured the output level of distortion products for several input levels of the fundamental frequency 250 Hz and reported that the second harmonic of the B71 becomes audible at an input level of 20 dB HL. However, for the B81 distortion is lower so that harmonics were found to be audible at a level of 30 dB HL of the fundamental frequency 250 Hz. At 125 Hz the amount of harmonic distortion is even higher than at 250 Hz for all transducers so that distortion products become audible at even lower presentation levels. Consequently, a deviation between the thresholds obtained at 125 Hz could be explained by differences in the distortion produced by the transducers, resulting in the lowest thresholds for the B71, followed by the B81, and the highest thresholds measured with the KH70. The influence of harmonic distortion on hearing thresholds was also assumed in prior work [10]. Nevertheless, this does not explain the deviations between the thresholds obtained with the different transducers at the high frequencies that were observed especially in the NH group.

Acoustic radiation is dominant for frequencies of 3000 Hz and higher and varies among different transducers. Frank and Crandell [12] measured the amount of acoustic radiation produced by the B71 and KH70 at 4000 Hz and observed that more acoustic radiation was produced by the B71 than by the KH70. A large level of acoustic radiation from the B71 at 6000 Hz was reported by Richter [13]. Also other results from the literature show, that acoustic radiation produced by BC transducers can contribute to the hearing sensitivity by BC and cause false high frequency air-bone gaps [14–17]. The B71 and B81 are both encapsulated in a plastic housing and likely to produce air-borne sound, whereas the KH70 is encapsulated in a rubber housing to guard against air-borne sound. Acoustic radiation could therefore be responsible for lower hearing thresholds measured with the B71 and B81 compared to the KH70 on NH participants at high frequencies. However, in the HI group no significant differences between the transducers were found for frequencies above 3000 Hz. Acoustic radiation from the transducers increases with increasing output levels, so that HI participants suffering from purely sensorineural hearing loss should be influenced by acoustic radiation in the same manner as NH participants. However, some of the participants from this study had a mixed hearing loss and it is reasonable to assume that the acoustic radiation was inaudible for them due to the conductive hearing loss. This may also explain why differences between the thresholds measured with the different transducers were not statistically significant for all frequencies above 3000 Hz in the HI group but it supports the argument that airborne sound is responsible for differences between the thresholds observed in the NH group.

Besides these deviations between the results with different transducers in the NH group, the BC hearing thresholds were found to be significantly lower than the AC hearing thresholds at 125 Hz, 3000 Hz, 4000 Hz, 6000 Hz and 8000 Hz. Generally, normal hearing does not require the AC thresholds to be the same as the BC thresholds. Studebaker [18] reported that the air-bone gap (AC threshold—BC threshold at a specific frequency) is a normally distributed variable with a mean of 0 dB and a standard deviation of 5 dB so that in subjects with normal middle-ear function the air-bone gap is not necessarily 0 dB. However, [19] examined distributions of air-bone gaps obtained by automated and manual audiometry and found that manual audiometry produced air-bone gaps that were not normally distributed showing evidence of biasing effects towards positive air-bone gaps due to assumptions of expected results. In this study the investigator was not influenced by previous audiograms, patient history, and AC and BC thresholds at other frequencies, because the transducers were connected to the sound field output of the audiometer so that the level shown on the audiometer did not correspond to the actual hearing level. The obtained thresholds levels had to be transformed into the real hearing levels after the experiment (see calibration section in the methods). Nevertheless, positive air-bone gaps indicated by significant differences between the AC and BC thresholds were obtained in the NH group at 125 Hz, 3000 Hz, 4000 Hz, 6000 Hz and 8000 Hz. Considering that the air-bone gaps are actually normally distributed [18], these significant differences can only be explained by differences between the transducers which influence the BC hearing thresholds, i.e., harmonic distortion at low and acoustic radiation at high frequencies.

## Conclusion

In this study significantly different BChearing thresholds were observed between the KH70 compared to the B71 and B81 at 125 Hz and higher frequencies such as 3000 Hz, 4000 Hz and 8000 Hz in NH and HI participants showing a poor reliability of thresholds obtained with different transducers. Results of audiometric BC threshold measurements should not be dependent on the type of transducer used in the test procedure. Therefore, the RETVFL-values used for the calibration of clinical audiometers should be reassessed and provided specifically for the different transducers. To obtain reliable new RETVFL values, the sample size has to be extended and the experiments should be conducted by a larger number of independent institutions.

The static force by which the transducers are coupled to the mastoid should be measured for each individual in those studies and the electroacoustic properties of each transducer (frequency response, harmonic distortion, maximum output) should be reported.

The differences between the results obtained with the different transducers found in this study are assumed to result from the different electro-acoustic properties of the transducers. In particular, the amount of harmonic distortion and the influence on BC hearing thresholds has to be further analyzed for the low frequencies 125 Hz and 250 Hz by measuring the exact output levels of the second harmonic at several fundamental frequency levels for the B71, B81 and KH70. Furthermore, the occurrence and behaviour of acoustic radiation of the different transducers B71, B81 and KH70 has to be further analyzed in future studies. For this, BC hearing thresholds need to be measured while the ear canal is occluded by an earplug to guard against airborne sound and again without occlusion. Normal hearing and hearing impaired participants who suffer from pure sensorineural hearing loss should be included in the study.

## Supporting information

S1 Table BC hearing thresholds measured with the B71, B81 and KH70 transducers. The table shows the BC hearing thresholds of all normal hearing and hearing impaired participants in this study (raw data). The thresholds were obtained with the B71, B81 and KH70 BC transducers.
(XLSX)
